# Impaired aortic strain and distensibility by cardiac MRI in children with chronic kidney disease

**DOI:** 10.1038/s41598-022-15017-9

**Published:** 2022-06-30

**Authors:** Donia M. Sobh, Ahmed M. Tawfik, Nihal M. Batouty, Hoda M. Sobh, Nashwa Hamdy, Ashraf Bakr, Riham Eid, Mohamed H. Awad, Basma Gadelhak

**Affiliations:** 1 Department of Diagnostic and Interventional Radiology, Mansoura University, Faculty of Medicine, 12 El-Gomhoreya Street, Mansoura, 35516 Egypt; 2 Department of Radiology, Andalusia Hospital AlShalalat, Andalusia Group for Medical Services, Alexandria, Egypt; 3 Department of Cardiology, Mansoura University, Faculty of Medicine, Mansoura, Egypt; 4 Pediatric Nephrology Unit, Department of Pediatrics, Mansoura University Children’s Hospital, Mansoura University, Faculty of Medicine, Mansoura, Egypt; 5 Department of Pediatrics, Mansoura University, Faculty of Medicine, Mansoura, Egypt

**Keywords:** Cardiology, Medical research, Nephrology, Cardiovascular diseases, Kidney diseases

## Abstract

Renal disease is associated with increased arterial stiffness. The aim was to investigate the effect of renal disease on regional aortic strain and distensibility in children with chronic kidney disease (CKD) by cardiac magnetic resonance imaging (MRI). The study included 30 children with CKD on hemodialysis, and ten healthy control subjects. Using cardiac MRI, maximal and minimal aortic areas were measured in axial cine steady state free precision images at the ascending aorta, proximal descending, and aorta at diaphragm. Regional strain and distensibility were calculated using previously validated formulas. Second reader aortic areas measurements were used to assess inter-observer agreement. Ascending aorta strain was significantly reduced in patients (38.4 ± 17.4%) compared to the control group (56.1 ± 17%), p-value 0.011. Ascending Aorta distensibility was significantly reduced in patients (9.1 ± 4.4 [× 10^−3^ mm Hg^−1^]) compared to the control group (13.9 ± 4.9 [× 10^−3^ mm Hg^−1^]), p-value 0.006. Strain and distensibility were reduced in proximal descending aorta and aorta at diaphragm but did not reach statistical significance. Only ascending aorta strain and distensibility had significant correlations with clinical and cardiac MRI parameters. Inter-observer agreement for strain and distensibility was almost perfect or strong in the three aortic regions. Aortic strain and distensibility by cardiac MRI are important imaging biomarkers for initial clinical evaluation and follow up of children with CKD.

## Introduction

The aorta is an elastic conduit that distends in systole and recoils in diastole, transforming pulsatile blood flow ejected from the left ventricle (LV) into continuous peripheral blood flow to maintain blood supply to the organs^1,2^. During systole, the aorta distends to accommodate almost 60% of the LV stroke volume. Part of the LV contractile energy is stored, and then released during diastole pushing the accommodated blood to the periphery^1^.

When the aorta becomes less distensible, the stored portion of LV stroke volume in the aorta during systole is decreased, and more blood volume is pushed to the peripheral circulation. Accordingly, systolic blood pressure is increased^1^. Decreased aortic distensibility is a determinant factor associated with vascular aging^3^. In addition, it seems to play an important role in the pathogenesis of hypertension and atherosclerosis and is associated with cardiovascular changes in diabetes and obesity^1,4^.

Chronic kidney disease (CKD) causes premature vascular aging^5^. Previous studies demonstrated that arterial stiffening is an early sign of cardiovascular disease in patients with CKD, and a promising marker for prediction of cardiovascular risk^6^.

Magnetic resonance imaging (MRI) is one of the methods for assessment of aortic strain and distensibility based on cross-sectional area measurements^2^. The advantages of MRI over echocardiography are better visualization of the aorta, and accurate measurements of cross-sectional area changes rather than one-dimensional wall diameter as seen in M-mode echocardiography^7^. Cardiac MRI is also the gold standard for assessment of LV dimensions and function, and arterial function assessment could be performed in the same investigation.

In children with CKD, previous reports showed that arterial stiffness contributes to cardiovascular morbidity or mortality^8^. In this context, arterial function is considered an important parameter in clinical evaluation of these patients^8^.

To our knowledge, the use of cardiac MRI for assessment of aortic function in pediatric CKD is poorly investigated. The aim of this study was to investigate aortic strain and distensibility by cardiac MRI in children with CKD.

## Methods

### Subjects

This prospective study was performed from January to April 2019. The study was approved by the institutional review board of Mansoura University, Mansoura, Egypt. The study was carried out in accordance to guidelines and regulations of medical research. Participants older than 16 years old, or guardians of younger children gave written informed consent.

Consecutive pediatric patients with stage 5 CKD on hemodialysis attending the pediatric nephrology outpatient clinic at our institution were recruited. The study population is part of a study project on multi-parametric cardiac MRI in children with CKD^9^.

The inclusion criteria were; age ≤ 18 years old, on chronic maintenance hemodialysis (3 sessions/ week for at least 3 months). Exclusion criteria included children with contraindications to MRI, and children with primary cardiovascular diseases. Ten healthy control children were recruited for pediatric CMR studies at our institution. Normally developed children with no known cardiovascular disease undergoing MRI for other indications were included. Participants older than 16 years old, or guardians of younger children gave written informed consent. Clinical and laboratory data were documented within 1 week of the cardiac MRI, including weight, height, body surface area (BSA), systolic blood pressure index (SBPi), hemoglobin (Hb), parathyroid hormone (PTH) level, serum calcium, phosphorus, alkaline phosphatase and dialysis adequacy (K dialyzer clearance of urea; t dialysis time; V volume of distribution of urea = Kt/V).

### Cardiac MRI

Cardiac MRI was performed on a 1.5-T scanner (Philips Ingenia, Best, Netherlands). Using retrospective ECG-gated steady-state free precession (SSFP) sequence, stack of contiguous slices was obtained in the axial, short axis, 3 and 4 chamber planes with the parameters; TR = 3.2–3.65 ms, TE = 1.6–1.83 ms, field of view (FOV) = 270 mm^2^, slice thickness = 5 mm, no slice gap.

### Post processing for ventricular volume and function

Images were transferred to separate workstation (extended MR 130 Workspace 2.6.3.5, Philips medical systems Netherland). Standard LV volumetric and functional analysis was performed using semi- automated method. Results were indexed to BSA.

### Image analysis

**Figure 1:**
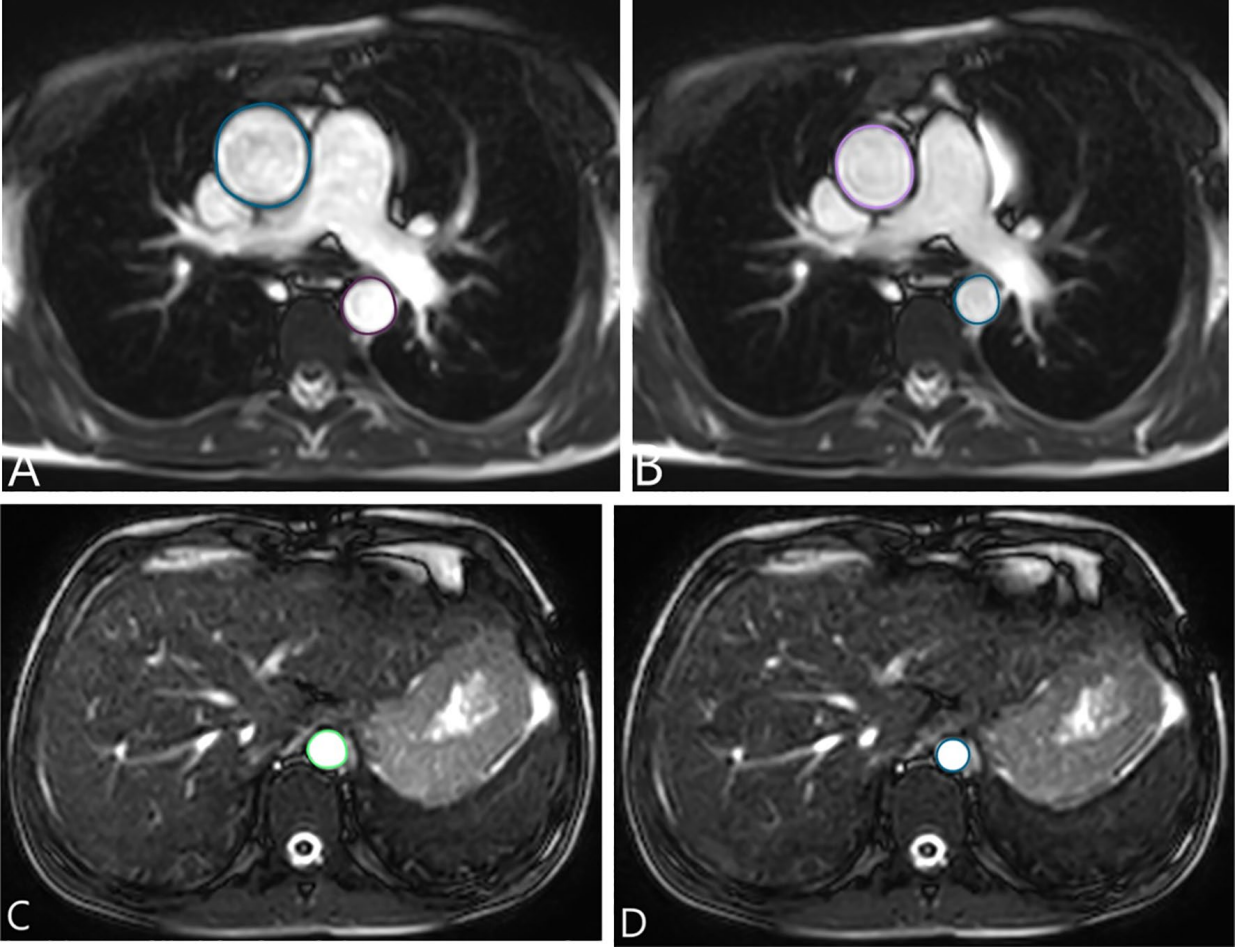
Axial cine SSFP images show measurement of maximal and minimal aortic area of ascending aorta (**A,B**), proximal descending aorta (**A,B**) and aorta at diaphragm (**C,D**).

The maximal and minimal aortic areas were obtained by manual tracing on axial cine SSFP images at three regions: the ascending aorta, proximal descending aorta, and aorta at diaphragm, Fig. 1a–d.

Distensibility was calculated according to the following formula: maximal aortic area − minimal aortic area/minimal aortic area × (systolic − diastolic blood pressure)^10^. Aortic strain was calculated as follows; maximal aortic area − minimal aortic area/minimal aortic area^7^. Maximal and minimal aortic areas and their absolute difference indexed to BSA were recorded.

### Statistical analysis

Statistical analysis was performed using IBM SPSS Version 22.0. Quantitative data were expressed as mean and standard deviation. Student t-test for parametric data and Mann Whitney test for non-parametric data were used to test the difference between patients and control groups. Correlations between variables were examined using Pearson or Spearman’s correlation coefficients. Multi-variable linear regression model was used to assess the effect of different independent variables on strain and distensibility. P values less than 0.05 were considered statistically significant. A posthoc power analysis revealed that a difference of 38% of the mean values of aorta distensibility between patients and control would have been detected with a high test power of more than 0.8.

## Results

### Participants’ characteristics and LV Cardiac MRI parameters

There were no significant differences between patients and control groups in age, gender, height, weight and BSA, Table 1. In the patients’ group, the duration of dialysis ranged between 2 and 110 months, median 16 months. The mean SBPi ± SD was 0.99 ± 0.13, mean Kt/V was 1.08 ± 1.42 mL/min, mean Hb was 10.1 ± 1.5 g/dl, PTH was 735 ± 465 ng/L, serum calcium was 8.5 ± 1.2 mg/dL, Phosphorus 4.5 ± 1.1 mg/dL, and alkaline phosphatase 727 ± 460 U/L.

**Table 1:** Baseline characteristics and LV parameters in the patients and control groups.

	Patients n = 30 Mean ± SD	Control n = 10 Mean ± SD	P value
Gender	15 male, 15 female	3 male, 7 female	0.2
Age (years)	13.8 ± 2.9	12.2 ± 2.8	0.138
Weight (kg)	32.2 ± 11.2	38.8 ± 8.6	0.111
Height (cm)	137 ± 15.7	141.9 ± 6.9	0.369
BSA (m^2^)	1.1 ± 0.24	1.2 ± 0.15	0.148
Systolic blood pressure (mmHg)	120.8 ± 14.8	117.7 ± 4.8	0.535
Diastolic blood pressure (mmHg)	77.7 ± 11.1	78.1 ± 2.1	0.907
Angiotensin converting enzyme inhibitor	15 (50%)		
Angiotensin receptor blocker	8 (27%)		
Calcium channel blocker	15 (50%)		
Alpha/beta blocker	4 (13%)		
LVEDVi (ml/m^2^)	111.8 ± 34	68.6 ± 7.8	0.000
Mi (g/m^2^)	75.5 ± 27.4	34.5 ± 6.3	0.000
LVEF (%)	59.6 ± 13.9	66.9 ± 3.4	0.011

Twenty patients received anti-hypertensive drugs (66%), 6 patients received only 1 drug (20%), 8 patients received 2 drugs (27%), 4 patients received 3 drugs (13%) and 2 patients received combination of 4 anti-hypertensive drugs (7%), listed in Table 1.

LV volume and mass were significantly higher and LV ejection fraction (EF) was reduced in patients group compared to control, Table 1.

### Aortic strain and distensibility in patients versus control

The maximal aortic and minimal aortic areas, their absolute differences, strain and distensibility of the three aortic regions are listed in Table 2.

**Table 2:** Comparison of cardiac MRI parameters between patients and control.

	Patients (n = 30)	Control (n = 10)	P value
**Ascending aorta**			
Maximal area indexed (mm^2^/m^2^)	599 ± 203.4	330 ± 57	0.000*
Minimal area indexed (mm^2^/m^2^)	448 ± 189	215.6 ± 56.6	0.000*
Absolute difference (mm^2^/m^2^)	151.6 ± 50.1	114.7 ± 24.8	0.04*
Strain (%)	38.4 ± 17.4	56.1 ± 17	0.011*
Distensibility (× 10^−3^ mm Hg^−1^)	9.1 ± 4.4	13.9 ± 4.9	0.006*
**Proximal descending aorta**			
Maximal area indexed (mm^2^/m^2^)	223.7 ± 63.1	137.3 ± 27.6	0.000*
Minimal area indexed (mm^2^/m^2^)	179.1 ± 57.8	104.5 ± 24.5	0.000*
Absolute difference (mm^2^/m^2^)	44.6 ± 24.7	32.8 ± 10.7	0.049*
Strain (%)	27 ± 14.9	33 ± 15.6	0.293
Distensibility (× 10^−3^ mm Hg^−1^)	6.3 ± 3.9	7.9 ± 4.2	0.252
**Aorta at diaphragm**			
Maximal area indexed (mm^2^/m^2^)	200.4 ± 74.2	125.7 ± 16.7	0.000
Minimal area indexed (mm^2^/m^2^)	147.7 ± 60.2	93.1 ± 18.2	0.000
Absolute difference (mm^2^/m^2^)	52.7 ± 24.1	32.5 ± 11	0.021*
Strain (%)	37.9 ± 14.4	36.8 ± 14.5	0.844
Distensibility (× 10^−3^ mm Hg^−1^)	8.7 ± 3.9	9.2 ± 3.6	0.730

#### a. Ascending aorta

Ascending aorta strain was significantly reduced in patients (38.4 ± 17.4%) compared to the control group (56.1 ± 17%), p-value 0.011. Similarly, aortic distensibility was significantly reduced in patients (9.1 ± 4.4 [× 10^−3^ mm Hg^−1^]) compared to the control group (13.9 ± 4.9 [× 10^−3^ mm Hg^−1^]), p-value 0.006.

#### b. Proximal descending aorta

There was no significant difference in strain between patients (27 ± 14.9%) and control group (33 ± 15.6%), p-value 0.29, as well no significant difference in distensibility between patients (6.3 ± 3.9 [× 10^−3^ mm Hg^−1^]) and control group (7.9 ± 4.2 [× 10 − 3 mm Hg − 1]), p-value 0.25.

#### c. Aorta at diaphragm

There was no significant difference in strain between patients (37.9 ± 14.4%) and control group (36.8 ± 14.5%), p-value 0.84, as well as no significant difference in distensibility between patients (8.7 ± 3.9 [× 10^−3^ mm Hg^−1^]) and control group (9.2 ± 3.6 [× 10^−3^ mm Hg^−1^]), p-value 0.73.

### Regional aortic strain and distensibility (Fig. 2a,b)

**Figure 2:**
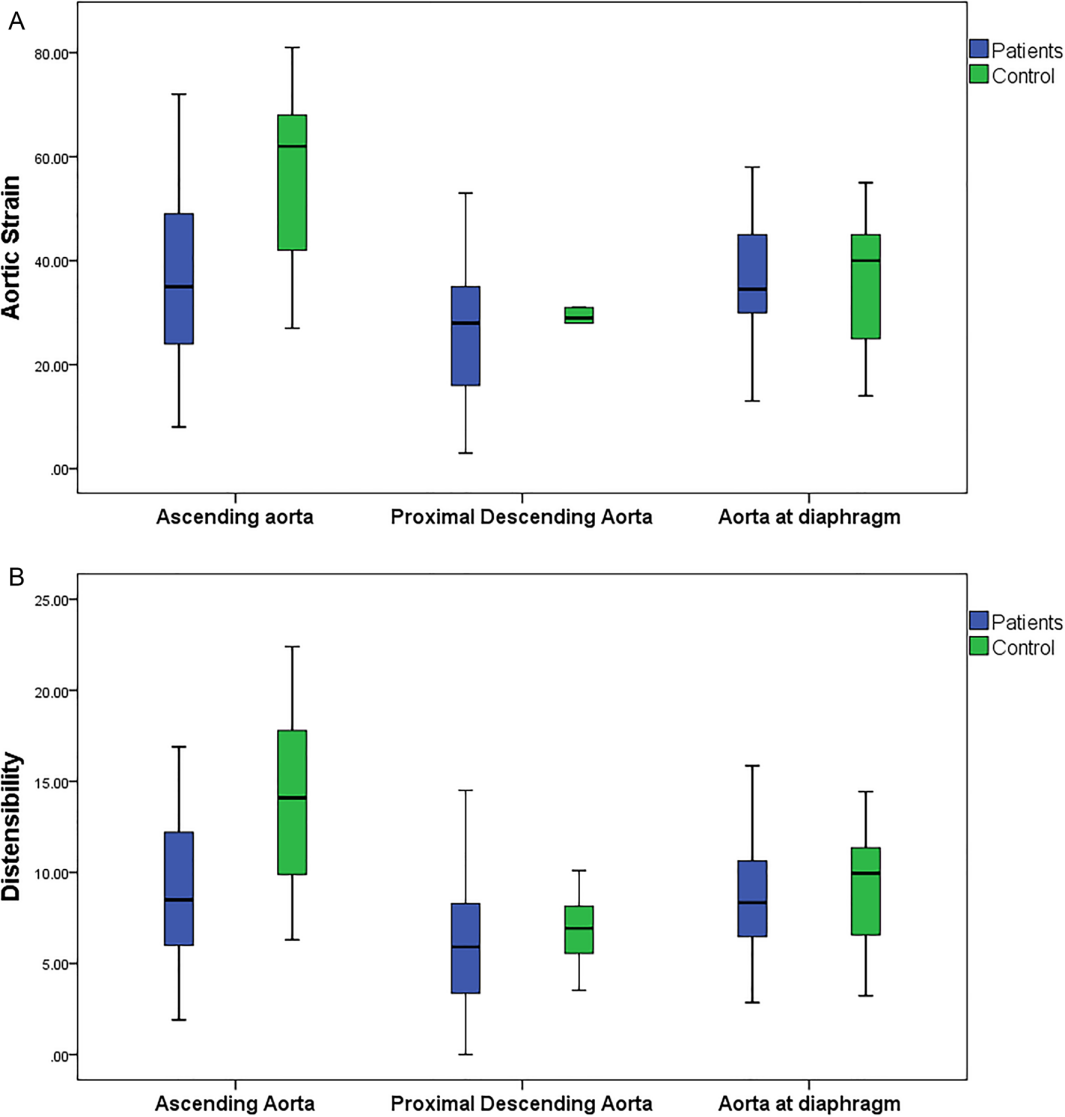
Box plots of aortic strain (A) and aortic distensibility (B) represent the difference between patients and control group at ascending aorta, proximal descending aorta and aorta at diaphragm.

In the patients’ group, the ascending aorta had significantly higher strain and distensibility than proximal descending aorta (p = 0.016, 0.020). Similarly In the control group, the ascending aorta had higher strain and distensibility than proximal descending aorta (p = 0.013, 0.011).

There was a trend for higher strain and distensibility in the ascending aorta compared to aorta at diaphragm in the patients’ group, (p = 0.99, 0.90). In the control group, aortic strain and distensibility in the ascending aorta were significantly higher than aorta at diaphragm (p = 0.040, 0.048).

The aorta at diaphragm had significantly higher strain and distensibility than proximal descending aorta in the patients’ group (p = 0.023, 0.049). In the control group, there was a trend for higher strain and distensibility in the aorta at diaphragm compared to proximal descending aorta (p = 0.88, 0.80).

### Association between aortic function and clinical and LV parameters

Ascending aorta distensibility had significant correlations with LV end diastolic volume indexed (EDVi) (r = −0.37, p = 0.046), LV mass indexed (Mi) (r = −0. 48, p = 0.008), SBPI (r = −0.378, p = 0.04) and Kt/v (r = −0.397, p = 0.03).

Multi-variable regression model showed that only Ktv had still statistically significant effect on distensibility after considering other co-factors (Beta = −0.402, P = 0.026).

Ascending aorta strain had significant correlation with LVEDVi (r = −0.399, p = 0.012), LVMi (r = −0.525, p = 0.001), and Kt/v (r = −0.385, p = 0.035). Multi-variable regression model showed that LVMi and Ktv had still statistically significant effect on strain after considering other co-factors (Beta = −0.488 and −0.416, P = 0.036 and 0.019).

The proximal descending aorta and aorta at diaphragm strain and distensibility were not correlated with cardiac MRI parameters or any other clinical parameter.

### Interobserver agreement for regional aortic strain and distensibility

Interobserver agreement analysis revealed almost perfect agreement for ascending aorta strain (ICC = 0.88, 95% CI 0.64–0.96) and distensibility (ICC = 0.91, 95% CI 0.75–0.97), strong agreement for proximal descending aorta strain (ICC = 0.75, 95% CI 0.25–0.92) and distensibility (ICC = 0.76, CI 0.31–0.92), and almost perfect agreement for aorta at diaphragm strain (ICC = 0.81, 95% CI 0.45–0.93) and distensibility (ICC = 0.81, 95% CI: 0.44–0.93).

## Discussion

The results of this study demonstrated decreased cardiac MRI-derived strain and distensibility of the thoracic aorta in pediatric patients with CKD compared to healthy controls.

In adult patients with CKD, similar findings were reported, and aortic stiffness worsened with aging^5,11,12^. Despite lower exposure of children with CKD to traditional cardio-vascular risk factors, and less effect of aging compared to adults; there is evidence that aortic stiffness contributes to increased risk of morbidity and mortality^8^. To our knowledge, this is the first study to report cardiac MRI-derived aortic strain and distensibility in children with CKD.

Cardiac MRI has the advantage of combined assessment of cardiac and aortic function in one investigation^6^. Unlike other methods for assessment of aortic stiffness, which measure the average stiffness of the whole vessel, cardiac MRI enables detection of more subtle regional changes in aortic stiffness, which may be isolated, or contribute to the stiffness of the vessel as a whole^6^.

The difference in strain and distensibility between patients and control was more evident in the ascending than the descending aorta. Regional variations in aortic distensibility may be explained by discrepancies in mechanical properties of the aorta along its length, which in turn is a function of the ratio between elastin and collagen. This ratio is highest in the proximal aorta and decreases gradually from proximal to distal. Therefore, the effect of different pathologic processes, which eventually cause fragmentation and destruction of elastin fibers; is not uniform along the thoracic aorta, with preferential stiffening more in proximal than distal aorta^5,13^.

Arteriosclerosis in CKD affects large vessels with reduced elastin and increased collagen in the medial layer, calcification, and hypertrophy of vascular smooth muscle cells^14,15^. Chronic kidney disease-mineral bone disorder plays an important role in development of vascular calcifications. Other factors that may contribute include anemia, endothelial dysfunction, neuro-hormonal activation and inflammation^6,14^.

Aortic stiffening exposes the LV to extra load, offsetting the balance between the heart and arterial system, or the ‘arterial-ventricular interaction’^6,15^. When the ascending aorta capacitance is impaired, the LV generates higher pressure to eject blood into the rigid arterial system, contributing to LV hypertrophy, dilatation, and eventually myocardial fibrosis^15,16^. Aortic stiffening and loss of its buffering function results in greater transmission of pressure fluctuations caused by ventricular contraction, exposing smaller arteries of the end-organs to higher systolic blood pressure. These changes eventually cause renal microvascular damage, contributing also to the progression of chronic renal disease^14,15,17^.

Arterial stiffening is an early sign of cardiovascular impairment in CKD, detectable before ventricular systolic dysfunction occurs^6,18^. Therefore, earlier and accurate quantification of aortic stiffness in renal patients potentially affects risk stratification, and is an interesting imaging biomarker for use in clinical studies and trials of novel treatment options for arterial stiffness^6^.

The current study, in line with previous studies, demonstrated that cross-sectional-derived strain and distensibility measurements by cardiac MRI have excellent reproducibility and are ideal for serial evaluations^2,7^. Aortic distensibility is defined as the blood volume change relative to a given pressure change. Because direct measurement of regional changes in blood volume is difficult, cross sectional area changes are alternatively estimated. Given that the axial length of arteries does not change significantly during expansion or recoil, changes in cross-sectional area could be assumed to represent changes in blood volume^4^.

Limitations of this study include the relatively small number of subjects, which may be the cause of weak correlations of arterial function parameters with clinical and laboratory data. The cross-sectional nature of the study did not allow for evaluating the effect of arterial function on prognosis or risk stratification.

In conclusion, thoracic aortic distensibility and strain are reduced in children with CKD compared to healthy controls. Cardiac MRI-derived aortic function is a potential imaging biomarker that could be used in initial clinical evaluation and follow up of children with CKD. Future studies are recommended to assess the effect of arterial stiffness on cardiovascular morbidity and mortality as well as assessment of response to available treatment options.

## Data Availability

The datasets used and analysed during the current study are available from the corresponding author on reasonable request.

## Abbreviations

BSA: Body surface area
CKD: Chronic kidney disease
EDVi: End diastolic volume indexed
FOV: Field of view
Hb: Hemoglobin
Kt/V: K dialyzer clearance of urea; t dialysis time; V volume of distribution of urea
LV: Left ventricle
Mi: Mass indexed
PTH: Parathyroid hormone
RV: Right ventricle
SBPi: Systolic blood pressure index
SSFP: Steady-state free precession
TE: Time of echo
TR: Time of repetition
